# A repository of microbial marker genes related to human health and diseases for host phenotype prediction using microbiome data

**Published:** 2019

**Authors:** Wontack Han, Yuzhen Ye

**Affiliations:** Computer Science Department, Indiana University, Bloomington, IN 47408, USA

**Keywords:** microbiome, microbial marker gene, type 2 diabetes, liver cirrhosis, immunotherapy efficacy

## Abstract

The microbiome research is going through an evolutionary transition from focusing on the characterization of reference microbiomes associated with different environments/hosts to the translational applications, including using microbiome for disease diagnosis, improving the efficacy of cancer treatments, and prevention of diseases (e.g., using probiotics). Microbial markers have been identified from microbiome data derived from cohorts of patients with different diseases, treatment responsiveness, etc, and often predictors based on these markers were built for predicting host phenotype given a microbiome dataset (e.g., to predict if a person has type 2 diabetes given his or her microbiome data). Unfortunately, these microbial markers and predictors are often not published so are not reusable by others. In this paper, we report the curation of a repository of microbial marker genes and predictors built from these markers for microbiome-based prediction of host phenotype, and a computational pipeline called Mi2P (from Microbiome to Phenotype) for using the repository. As an initial effort, we focus on microbial marker genes related to two diseases, type 2 diabetes and liver cirrhosis, and immunotherapy efficacy for two types of cancer, non-small-cell lung cancer (NSCLC) and renal cell carcinoma (RCC). We characterized the marker genes from metagenomic data using our recently developed subtractive assembly approach. We showed that predictors built from these microbial marker genes can provide fast and reasonably accurate prediction of host phenotype given microbiome data. As understanding and making use of microbiome data (our second genome) is becoming vital as we move forward in this age of precision health and precision medicine, we believe that such a repository will be useful for enabling translational applications of microbiome data.

## 1. Introduction

Recent studies of microbiomes (i.e., communities of microorganisms) have shaped a new view of the biological world in which various microbial organisms play important roles in the health of humans, animals, plants, and the environment.^1–4^ Metagenome-wide association studies^5^ have enabled the high-resolution discovery of associations between the microbiome and human diseases, including type 2 diabetes,^6^ liver cirrhosis,^7^ atherosclerotic cardiovascular disease,^8^ colorectal cancer^9^ and rheumatoid arthritis.^10^ The announcement of the National Microbiome Initiative (NMI) on May 13, 2016, marks a milestone in microbiome research. The NMI aims to advance the understanding of microbiome behavior and enable protection and restoration of healthy microbiome function. Development of computational tools for interpretation and integration of meta-omics data will be key to advancing the field and ultimately achieving the goal of the NMI.

Unlike traditional microbial genomic sequencing projects, metagenomics attempts to directly characterize the entire collection of genes within an environmental sample (i.e., the metagenome) and analyze their biochemical activities and complex interactions.^11,12^ Landmark progress in metagenomics occurred in 2004^13,14^ when two research groups published results from large-scale environmental sequencing projects. Many more metagenomic projects have been conducted or are ongoing, representing broadened applications from ecology and environmental sciences^15^ to the chemical industry^16^ and human health.^17^ Metagenomics, in principle, enables the study of any microbial organism, including the large number of microorganisms that cannot be isolated or are difficult to grow in a lab. More importantly, microbes, by nature, live in communities where they interact with each other by exchanging nutrients, metabolites, and signaling molecules. Metagenomics enables the characterization of microbes in natural environments, addressing important biological questions related to microbial environments such as the diversity of microbes in different environments,^18^ microbial (and microbe-host) interactions,^19^ and the environmental and evolutionary processes.^20^

Earlier metagenomics studies focused on the characterization of reference microbiomes associated with different environments/hosts. Recent studies shift the emphasis to the translational applications, including using microbiome for disease diagnosis, improving the efficacy of cancer treatments (including cancer chemotherapy and immunotherapy), and prevention of diseases (e.g., using probiotics).^21^ Gut bacterium *Eggerthella lenta* was found to be able to manipulate cardiac drug inactivation.^22^ Harnessing the host immune system constitutes a promising cancer therapeutic because of its potential to specifically target tumor cells while limiting harm to normal tissues. Recent clinical success has fueled the enthusiasm about immunotherapy using antibodies that block immune inhibitory pathways, specifically, the CTLA-4 and the PD-1/PD-L1 axis.^22,23^ The gut microbiota plays an important role in shaping hosts immune responses,^24^ so there is no surprise that a few recent studies have shown that intestinal microbiota (and some particular microbial species/strains) can mediate immune activation in response to chemotherapeutic agents and immunotherapy. Sivan and colleagues^25^ found that commensal Bifidobacterium promotes antitumor immunity and facilitates anti PD-L1 efficacy. They also found that oral administration of Bifidobacterium alone improved tumor control to the same degree as anti PD-L1 therapy (checkpoint blockade), and combination treatment nearly abolished tumor outgrowth. Gut microbiota can also modulate the actions of chemotherapeutic drugs used in cancer and other disease, reducing the toxicity of chemotherapeutic compounds and improve their efficacy.^26^ A working knowledge of the micro-biome (our second genome^27^) is vital as we move forward in this age of precision health and precision medicine,^28^ especially in the area of cancer research, which aims at effective treatments for various kinds of cancer based on the knowledge of genetics, biology of the disease and host-microbiome interactions.^29^

The success of the translational applications of microbiome data relies on the characterization of differential markers (species, genes, biological pathways, among others) that can differentiate different groups of microbiome data (e.g., healthy individuals versus patients, treatment responders versus non-responders). It is also important to understand factors influencing the gut microbiome and strategies to manipulate the microbiome to augment therapeutic responses and disease prevention.^30^

To derive microbial markers that are associated with a specific host phenotype (e.g., healthy versus diseased), a key task is to compare two groups of microbiome (e.g., one group of microbiome data derived from healthy individuals versus a group derived from patients) to detect *consistent* differences (e.g., species or genes) between the groups, considering the large inter- and intra-individual variations of the microbiome.^31^ The typical analysis workflow is to assemble and annotate metagenomic datasets individually or as a whole, followed by statistical tests to identify differentially abundant species/genes. The subtractive assembly approaches we previously developed, subtractive assembly (SA)^32^ and concurrent subtractive assembly (CoSA) approach,^33^ are *de novo* assembly approaches for comparative metagenomics that first detect differential reads between two groups of metagenomes and then only assemble these reads. When evaluated using simulated and real type 2 diabetes microbiome datasets,^33^ our subtractive assembly approaches reduce the datasets up front, which also result in better characterization of the differential genes.

Recent studies have revealed microbial markers for disease diagnosis and other purposes, and predictors built based on these markers have achieved promising accuracy for predictions. The pitfall of most of these studies is that the microbial markers and predictors built from these markers are not made available for others to use. For example, Qin et al.^7^ constructed a support vector machine discriminator based on microbiome data for liver cirrhosis prediction using 15 gene markers, achieving impressive accuracy, with AUC (area under the receiver operating characteristic curve) of 0.918 and 0.838, respectively, for training and leave-one-out cross-validation. Although the authors listed the identities of these 15 genes in a supplementary table (Supp Table 12 in^7^), they did not release the gene sequences, nor the discriminator they built. It makes it impossible for others to use their marker genes and predictors. Using our recently developed computational approach CoSA,^33^ we re-analyzed several large collections of publicly available microbiome datasets, in an attempt to create a repository of microbial marker genes and the predictors built from these marker genes for translational applications of microbiome data (e.g., to predict if a cancer patient is likely to be responsive to PD-1 blockage treatment given his/her microbiome data). We note there is no shortage of microbiome repositories; instances include the Human Microbiome Project repository (http://hmpdacc.org) and the MG-RAST server (https://www.mg-rast.org). However, there is no repository of bacterial marker genes and predictors for microbiome-based predictions to the best of our knowledge. As a proof of concept, we focused on two diseases, type 2 diabetes and liver cirrhosis, and two types of cancers. We first extracted microbial marker genes from these microbiome datasets, then built predictors using these genes, and finally created a repository of the marker genes and predictors, as well as a companion computational pipeline for using this repository.

## 2. Methods

### 2.1. Microbiome datasets

We focus on microbial genes related with two diseases and the treatment efficacy of two types of cancer:

- T2D (type 2 diabetes). We used the T2D cohort from a study,^6^ which contains microbiome data from two groups of 70-year-old European women, one group of 50 with T2D and the other a matched group of healthy controls (NGT group; 43 participants). We previously used this cohort for testing our subtractive assembly approaches.^32,33^
- Cirrhosis (liver cirrhosis). Qin et al.^7^ derived metagenomic datasets from 98 Chinese patients with liver cirrhosis and 83 healthy individuals as training datasets to infer marker genes and build a predictor, and microbiome data from additional 25 patients and 31 healthy controls as validation datasets. Similarly, we used their training datasets for characterization of marker genes and training of predictors, and their validation datasets for independent tests of the predictors for liver cirrhosis.
- NSCLC (non-small-cell lung cancer). It has been shown that gut bacteria can affect patient responses to cancer immunotherapy (e.g., immune checkpoint inhibitors ICIs that target the PD-1/PD-L1 axis). Routy et al.^34^ found that primary resistance to ICIs can be attributed to abnormal gut microbiome composition, and fecal microbiota transplantation (FMT) from cancer patients who responded to ICIs into germ-free or antibiotic-treated mice ameliorated the antitumor effects of PD-1 blockade, whereas FMT from non-responding patients failed to do so. They sequenced the microbiome of the stool samples at diagnosis, and showed correlations between clinical responses to ICIs and relative abundance of *Akkermansia muciniphila*. We used microbiome datasets from this study, which includes 32 non-responders and 33 responders, aiming to infer marker genes that can be used to distinguish responders from non-responders.
- RCC (renal cell carcinoma). We used datasets from the same study^34^ that involve 20 non-responders versus 42 responders to a different cancer type, renal cell carcinoma.

Table 1 summarizes the microbiome datasets that were re-analyzed in this paper.

### 2.2. Microbial gene characterization and quantification

For each collection of above mentioned microbiome datasets, we first applied CoSA to assemble genes that are potentially differential between the groups (i.e., for the T2D collection and the liver collection, the patient group versus group of healthy individuals, and for the NSCLC and RCC collections, responders versus non-responders). These genes were then subject to feature selection. Using selected marker genes, different machine learning (ML) approaches were employed to build predictors for microbiome-based host phenotype prediction. We refer the readers to our previous publications^32,33^ for details about our subtractive assembly approach CoSA. Briefly, the CoSA approach uses a Wilcoxon rank-sum (WRS) test to detect k-mers that are differentially abundant between two groups of microbiomes (CoSA uses KMC2^35^ for k-mer counting, and employs the “mannwhitneyutest” function from ALGLIB (http://www.alglib.net) for the test). It then uses identified differential k-mers to extract reads (by a voting strategy) that are likely from the sub-metagenome with consistent abundance differences between the groups of microbiomes. Further, CoSA attempts to reduce the redundancy of reads (from abundant common species) by excluding reads containing abundant k-mers. Extracted reads are then assembled using MegaHit,^36^ and genes are predicted from the assembled contigs using FragGeneScan.^37^ The quantification of the genes in each microbiome is done by reads mapping of shotgun reads onto the genes using Bowtie 2.^38^ We counted a gene’s abundance based on the counts of both uniquely and multiplely mapped reads (the contribution of multiplely mapped reads to a gene was computed according to the proportion of the read counts divided by the gene’s unique abundance^7^). The read counts were then normalized per kilobase of gene per million of reads in each sample.

### 2.3. Inference of microbial marker genes using machine learning approaches

**Fig. 1:**
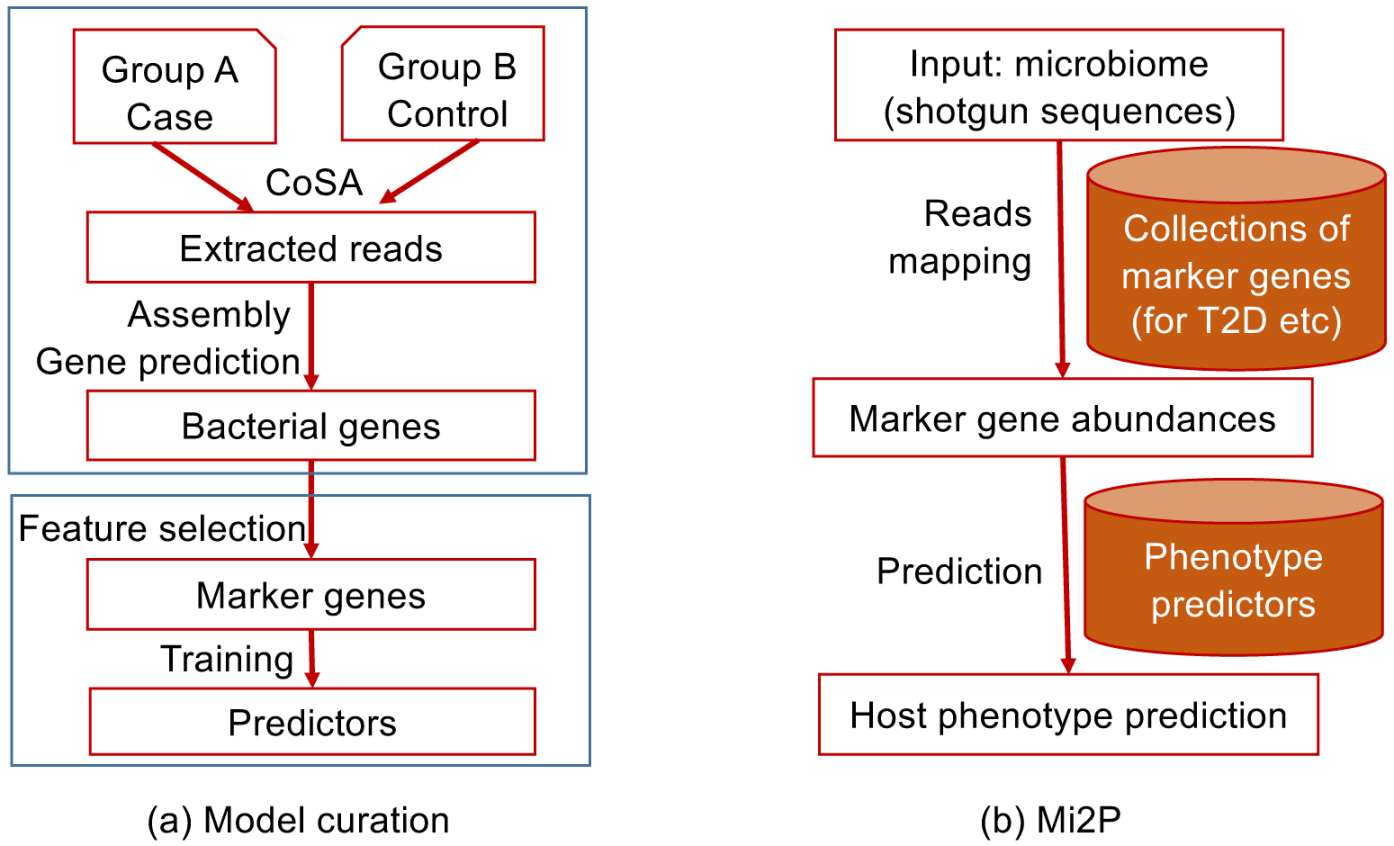
Schematic representations of the model curation based on CoSA (a) and Mi2P (Microbiome to Phenotype) pipeline (b).

Microbial genes assembled and quantified mentioned above for the different microbiome datasets were used as candidate features for selecting microbial marker genes and for training predictors for microbiome-based host phenotype prediction (see Figure 1(a)). For feature selection, we first applied a q-value cutoff and then used two different feature selection methods (tree-based feature selection and L1-based feature selection) to select a smaller number of microbial genes, and used them as microbial marker genes. We tried different ML algorithms for phenotype prediction, including Support Vector Machines (SVM), Random Forests (RF), Decision Trees (DT), Neural Networks (NN), and K-nearest Neighbor (KN) approach, along with different cross-validation strategies. We used the scikit-learn (http://scikit-learn.org) implementation of these ML approaches in this study. We tested RF with 10, 100 and 1000 trees and KN with 20 neighbors. For NN, we used Bernoulli Restricted Boltzmann Machine (RBM) with 3200 binary hidden units. We used the default settings for SVM and DT.

### 2.4. Mi2P: from microbiome to phenotype

We created a repository of above mentioned microbial marker genes and predictors built from the marker genes. We also developed a computational pipeline called Mi2P (which stands for “from Microbiome to Phenotype”) for users to use this repository. As shown in Figure 1(b), Mi2P is composed of three main steps: 1) mapping of metagenomic sequencing reads onto the marker genes using Bowtie 2;^38^ 2) quantification of the marker genes based on read counts, using both uniquely and multiplely mapped reads (see 2.2); and 3) the estimated gene abundances are used as input features to the microbiome-based phenotype predictors. A wrapper script is included in the pipeline for the one-stop use of our pipeline, which takes a metagenomic dataset as the input, and reports prediction as the main output. It also outputs some intermediate results including the estimated gene abundances. Mi2P is available as open source software for download at sourceforge (https://sourceforge.net/projects/mi2p/).

## 3. Results

### 3.1. Accuracy of microbiome-based predictors

We built predictors for predicting host phenotype based on the microbiome data. We evaluated the accuracy of the predictors using different cross-validation strategies and ML approaches. Furthermore, we tested two different feature selection approaches (tree-based and L1-based) with liver cirrhosis data sets. Since we have already reported the performance of T2D prediction in our previous publications,^32,33^ we focused on reporting the results for liver cirrhosis and cancer treatment responsiveness prediction based on microbiome data in this paper.

**Fig. 2:**
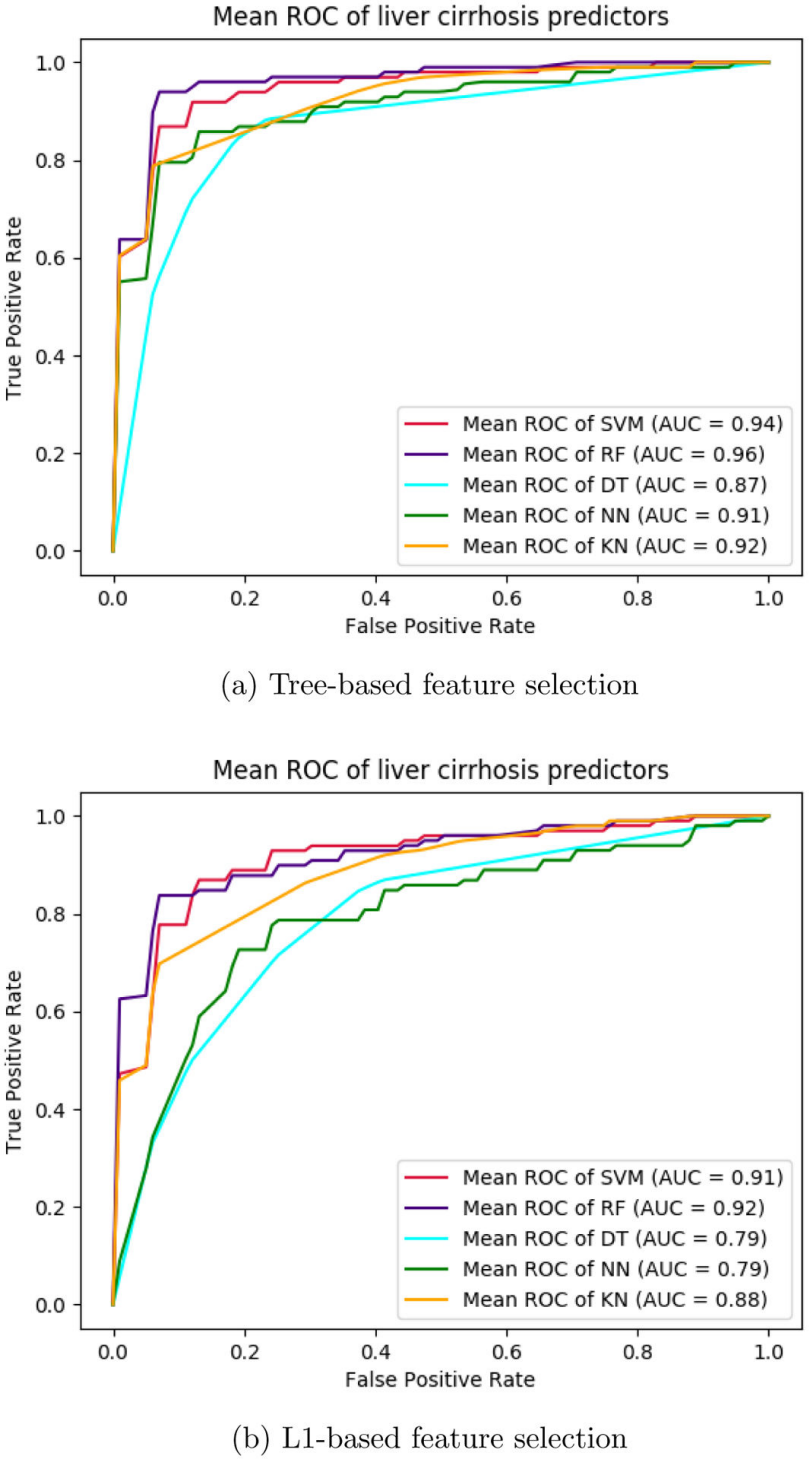
Receiver operating characteristic (ROC) plots of the liver cirrhosis predictors using different ML approaches. We also tested two feature selection methods: tree-based feature selection and L1-based feature selection, and the results are shown in (a) and (b), respectively. The ROC curves were averaged over five cross validation results.

Figure 2 shows the ROC plots for liver cirrhosis prediction using different ML approaches and feature selection methods. The figure shows that RF achieved better predictions than SVM approach. It also shows that predictors built from genes selected using the tree-based feature selection method performed better as compared to L1-based feature selection method. We therefore chose the tree-based feature selection as the default approach in our pipeline.

Table 2 summarizes the accuracy of the predictors we built for liver cirrhosis. Our SVM based predictor achieved comparable performance as the predictor reported in Qin et al..^7^ However, our RF based predictor achieved significantly better predictions with higher AUCs. We speculate that the accuracy improvement is a result of the combination of more marker genes and a different machine learning approach (RF). We note that we tested RF using different numbers of trees, including 10, 100 and 1000. We found that RF with 100 trees and 1000 trees achieved slightly better predictions than RF with 10 trees. Balancing running time and accuracy, we chose RF with 100 trees.

Table 3 summarizes the accuracy for predicting immunotherapy responders versus nonresponders based on microbiome data. Correlations between clinical responses to immunotherapy (ICI) and the relative abundance of *Akkermansia muciniphila* were reported in,^34^ however, no predictors were built by the authors. Here, we built predictors for immunotherapy responsiveness using the RF approach with a small collection of marker genes, which achieved reasonably accurate predictions for NSCLC. Predictions of RCC based on microbiome data were less accurate. We tested RF predictors with different trees, and results show that RF with 100 trees performed relatively well for both cancers, similar to prediction of liver cirrhosis. Therefore, we chose RF predictors with 100 trees for immunotherapy resposiveness prediction to include in our Mi2P package. We note that we also applied SVM approach to this dataset, which however achieved much worse predictions (AUC = 0.61) than the RF predictors.

### 3.2. Microbial marker genes

We include the sequences of microbial marker genes (both proteins and gene sequences), along with their annotations (by hmmscan^39^) in the Mi2P package. Table 4 shows a few examples identified from the liver cirrhosis cohort. These marker genes can be either more abundant in healthy individuals (i.e., depleted in liver cirrhosis microbiomes), or more abundant in liver cirrhosis microbiomes. We also note that four out of 41 (10%) enriched genes in liver cirrohosis microbiomes have no functional annotations.

### 3.3. Running time of Mi2P pipeline

We provide a wrapper script in Mi2P pipeline for users to employ our repository of microbial marker genes and predictors. We show that this pipeline gives fast prediction of host phenotype from a query microbiome dataset (of shotgun sequences), thanks to the relatively small number of microbial marker genes that need to be considered. For example, on a linux computer (with Intel(R) Xeon(R) CPU E5–2623 v3 @ 3.00GHz), running the pipeline for two test datasets, one from the liver cirrhosis collection (ERR528314 with 3 Gbps), and the other one from the NSCLC collection (ERR2213736 with 2 Gbps) each took less than 6 min to complete.

## 4. Discussion

Our current repository of microbial marker genes and predictors is rather limited, covering only four host phenotypes. We plan to apply the same analysis to more collections of microbiome datasets associated with human diseases and treatment efficacy. We believe there will be no shortage of such datasets due to the soaring interests in microbiome research associated with human health and diseases. In addition, we will seek to collect microbial marker genes using other approaches (e.g., based on the literature search) to enrich our repository.

A challenging problem in making our repository of microbial maker genes and predictors useful will be the generalization issue, due to both the biological complexity (e.g., stratification of the samples that were used to build the classifiers) and technical complexity (e.g., over-fitting of the predictors). The generalization issue is a general problem in machine learning, and methods have been proposed to alleviate the problem. We will explore some of the existing approaches to address this challenge. In addition, we will explore approaches to provide confidence of predictions, rather than to simply provide yes or no prediction.

Further studies of the microbial marker genes will be needed to understand why they are important for microbiome-host interaction, contributing to the host phenotype. We also note that a significant fraction of the identified marker genes are of unknown functions. We will exploit different homology- and context-based approaches to predict the functions of these genes. Boosted by the accumulation of microbial genomes and metagenomes, a few new methods, including our own guilt-by-association approach (the community profiling approach), have been developed for functional annotation of microbial genes.^40,41^ We plan to utilize these approaches in our future research.

## Figures and Tables

**Table 1:** Summary of the microbiome datasets for training the predictors.

Abr.	Disease	Reference	# of samples	Total base pairs
T2D	Type 2 diabetes	[6]	93	225 GB
Cirrhosis	Liver cirrhosis	[7]	181	817 GB
NSCLC	Non-small-cell lung cancer	[34]	65	153 GB
RCC	Renal cell carcinoma	[34]	62	147 GB

**Table 2:** Accuracy of microbiome-based predictors for liver cirrhosis.

	methods	# of marker genes	SVM	RF (100 trees)	NN	KN
Cross^a^	Qin et al.	15^c^	0.84^c^	N/A	N/A	N/A
	Our approach	46	0.92	0.92	0.88	0.71

Validation^b^	Qin et al.	15^c^	0.84^c^	N/A	N/A	N/A
	Our approach	46	0.83	0.93	0.81	0.72

a: the “cross” columns show the leave-one-out validation result (see Figure 2 (a) for 5 fold cross-validation results).

b: validation using microbiome data unseen in the training of the predictor.

c: numbers taken from the paper.^7^

**Table 3:** Accuracy of microbiome-based prediction of responders versus non-responders to cancer treatment using RF (with 10, 100, and 1000 trees), DT and NN approaches.

Cancer type	# of marker genes	RF			DT	NN
		10	100	1000	mean AUC	mean AUC
NSCLC	116	0.86	0.91	0.89	0.72	0.81
RCC	85	0.84	0.83	0.81	0.79	0.78

**Table 4:** Examples of microbial marker genes for liver cirrhosis prediction.

Depleted in liver cirrhosis microbiome		
H_k99_23554_31_534_−	Tripartite ATP-independent periplasmic transporters	DctQ
H_k99_23763_1365_1613_−	Helix-turn-helix domain	HTH_31
H_k99_38620_1_453_+	Acyltransferase family	Acyl_transf_3
H_k99_59586_373_654_−	Amidohydrolase	Amidohydro_2
H_k99_64410_1_617_−	REC lobe of CRISPR-associated endonuclease Cas9	Cas9_REC
Enriched in liver cirrhosis microbiome		
L_k99_1592_1_390_−	Polysaccharide biosynthesis C-terminal domain	Polysacc_synt_C
L_k99_7366_1_565_−	Carbon starvation protein CstA	CstA
L_k99_13622_1_326_+	Septation ring formation regulator, EzrA	EzrA
L_k99_52773_82_623_+	Sodium:sulfate symporter transmembrane region	Na_sulph_symp
L_k99_52825_1_408_+	D-isomer specific 2-hydroxyacid dehydrogenase	2-Hacid_dh_C
